# A New Protein–Ligand Trapping System to Rapidly Screen and Discover Small-Molecule Inhibitors of PD-L1 from Natural Products

**DOI:** 10.3390/molecules30081754

**Published:** 2025-04-14

**Authors:** Yazhuo Huang, Senfeng Sun, Runxin Yin, Zongtao Lin, Daidong Wang, Wanwan Wang, Xiangyu Fu, Jing Wang, Xinyu Lei, Mimi Sun, Shizhong Chen, Hong Wang

**Affiliations:** 1 School of Pharmaceutical Sciences, Peking University, Beijing 100191, China; hyztime@126.com (Y.H.); wangjing1988@bjmu.edu.cn (J.W.);; 2 Department of Chemistry, University of Pennsylvania, Philadelphia, PA 19104, USA; 3 School of Pharmacy, Shanxi University of Chinese Medicine, Xianyang 712046, China

**Keywords:** Chinese herbal medicines, inhibitors, protein–ligand trapping (PLT) system

## Abstract

Chinese herbal medicines have played a significant role in the development of new and effective drugs, but how to identify the active ingredients from complex extracts of traditional Chinese herbal medicines was a research difficulty. In recent years, few studies have focused on high-efficiency identification of small-molecule inhibitors of Programmed Death Ligand 1 with lower antigenicity and flexible structure tunability. In order to identify small molecule inhibitors of PD-L1 from complex Chinese herbal extracts, this study established a protein–ligand trapping system based on high-performance liquid chromatography coupled with a photo-diode array detector, ion trap/quadrupole time-of-flight tandem mass spectrometry, and a Programmed Death Ligand 1 affinity chromatography unit (ACPD-L1-HPLC-PDA-IT-TOF (Q-TOF)-MS) to rapidly screen and identify small-molecule inhibitors of Programmed Death Ligand 1 from *Toddalia asiatica* (L.) Lam. Fourteen components were then identified as PD-L1 binders, and surface plasmon resonance (SPR) validation results showed that six of them—magnoflorine (**6**), nitidine (**22**), chelerythrine (**24**), jatrorrhizine (**13**), toddaculin (**68**), and toddanol (**45**)—displayed PD-L1 binding activity. Laser scanning confocal microscopy results demonstrated that these compounds effectively inhibited the binding of PD-1 to PD-L1 in a dose-dependent manner. Additionally, flow cytometry analysis indicated they could promote human lung cancer cell line (A549) apoptosis when co-cultured with Peripheral Blood Mononuclear Cells (PBMCs). The system’s innovation lies in its first integration of dynamic protein–ligand trapping with multi-dimensional validation, coupled with high-throughput screening capacity for structurally diverse natural products. This workflow overcomes traditional phytochemical screening bottlenecks by preserving native protein conformations during affinity capture while maintaining chromatographic resolution, offering a transformative template for accelerating natural product-derived immunotherapeutics through the PD-1/PD-L1 pathway.

## 1. Introduction

During the past few decades, cancer immunotherapy has achieved resounding progress. In fact, Science in 2013 described it as an extraordinary paradigm shift in oncotherapy [1]. Cancer immunotherapies consist of cancer vaccines [2], adoptive cell therapy with tumor-infiltrating lymphocytes [3], chimeric antigen receptor T cell (CAR-T) therapy [4], and immune checkpoint blockade therapy [5,6]. Among diversiform immunotherapies, immunotherapy strategies to block inhibitory immune checkpoints have attracted significantly considerable attention. Immune checkpoints are negative regulators that regulate immune homeostasis between costimulatory and coinhibitory signals, which in turn protect the immune system from damage during an exaggerated autoimmune response driven by proliferating immune cells, such as programmed death 1 (PD-1), programmed cell death ligand 1 (PD-L1), cluster of differentiation 28 (CD28), T cell immunoglobulin domain and mucin domain-3 (TIM-3), and galectin-9 (Gal-9) [7]. However, the overactivation of intrinsic immune checkpoint mechanisms in a tumor microenvironment could promote the regression or even death of specific immune cells and activate co-inhibitory signaling pathways, leading to immune evasion of tumor issues from immune responses [8,9].

PD-1, a member of the CD28 family, is primarily expressed by activated T cells, B cells, dendritic cells (DCs), regulatory T cells (Tregs), and tumor-associated macrophages (TAMs) [10,11]. PD-1 can be activated by its specific ligand, PD-L1, which is commonly expressed on the surface of antigen-presenting cells (APCs) and kinds of tumor cells [12,13], while PD-L2 expression is restricted and barely found in tumor cells [14]. In the tumor microenvironment, overexpressed PD-L1 is capable of interacting with PD-1 on sensitized immune cells, inducing T cell proliferation suppression and the production of cytokines, such as interferon-γ (IFN-γ) and interleukin 2 (IL-2). This process could ultimately result in cytotoxic T-cell dysfunctions and achieve tumor cell evasion from immune surveillance [15,16].

Blockades of PD-1 and PD-L1 have demonstrated unprecedented tumor suppressive function in clinical practice [17]. In the last decade, the FDA has approved six monoclonal antibodies (mAbs) targeting PD-1 or PD-L1. These macromolecular antibody-based drugs are applied to do melanoma remedy [18], non-small-cell lung cancer [19], ovarian cancer [20], bladder cancer [21], and hematological malignancies [22,23]. Antibody-based drugs are simultaneously controversial on account of many inherent characteristics, such as hard-adjusted pharmacokinetic characteristics, limited tissue and tumor penetration, poor oral bioavailability, immunogenicity [24,25], induction of drug resistance, low compliance, and expensive production [26,27]. Thus, an increasing number of studies are focusing on modifying the drug configuration to avoid the disadvantage of mAbs and develop small-molecule suppressive modulators as potential supplements or alternatives, which can offer the convenience of highly compliant medication methods, more controllable physiochemical and toxic profiles [28], improved response rates, and affordability [29]. The active compound structures that have demonstrated a PD-1/PD-L1 inhibiting function contain cyclic peptides, sulfonamide, biphenyl, bromine-substituted benzyl ether, and pyridinamide derivatives [30]. However, only one oral small-molecule inhibitor of PD-1/PD-L1, CA-170, has entered a phase I clinical trial for treating progressive solid tumors and lymphomas [31]. Meanwhile, a lack of rapid strategies has created obstacles to the identification and discovery of effective low-molecular-weight immunomodulators. Moreover, there is an urgent need to develop a single productive approach for the rapid identification of small-molecule PD-1/PD-L1-targeted therapeutics. Many natural products have been reported as inhibitors against various proteins; for instance, chlorogenic acid acts as an α-glucosidase inhibitor [32], nitidine targets topoisomerase I [33], and chelerythrine inhibits protein kinase C [34]. PD-L1 inhibitors have better clinical benefits and lower toxicity than PD-1 inhibitors. Therefore, there is a growing need for natural product-based inhibitors targeting PD-L1. The present study established a novel PLT system to rapidly discover pre-compounds for small-molecule inhibitors of PD-L1 from traditional Chinese medicine (TCM), which is rich in small-molecule natural products with unique structures. Specifically, this PLT system was used to distinguish PD-L1-targeted micromolecules in *Toddalia asiatica* (L.) Lam (TA), a traditional Miao herb that is rich in abundant compounds, such as coumarins [35,36], alkaloids [37], terpenoids [38], flavonoids [39], and lignans [36]. TA, which has shown significant potential for anti-tumor effects [40]; hemostatic and procoagulant effects; and anti-inflammatory [41], antioxidant [42], and antibacterial [43] properties in modern pharmacological research, has the potential to contain PD-L1 inhibitors with great drug-like properties. Ultimately, the trapping system screened 14 potential PD-L1-inhibiting small-molecule compounds in TA based on AC_PD-L1_-HPLC-PDA-IT-TOF (Q-TOF)-MS. Six of these compounds were verified using SPR and cytopharmacology, which may expedite the discovery of lead compounds for suppressing PD-1 and PD-L1.

## 2. Results and Discussion

### 2.1. The Effectiveness of the New PLT System Validated by Positive Medicine

**Figure 1:**
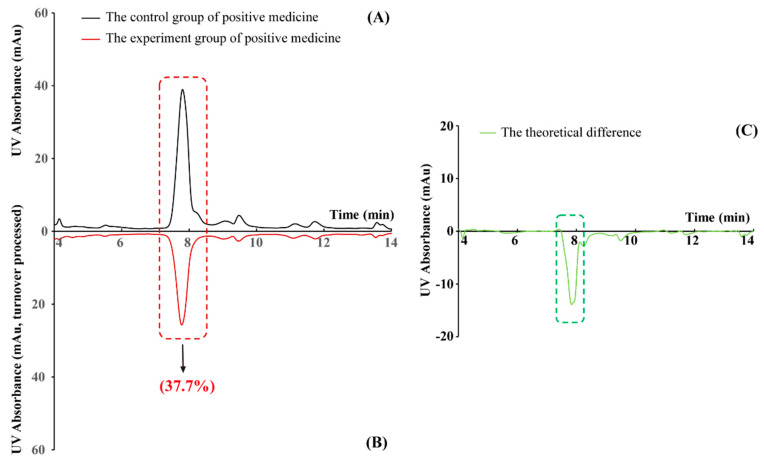
Graphical results of positive medicine. (**A**) HPLC chromatogram of the blank group of HQG; (**B**) HPLC chromatogram of the experimental group of HQG (turnover processed). (**C**) Theoretical difference in control group and experimental group of positive medicine. The peaks in the red frame are the UV absorption of positive medicine; RCPs in the parentheses show the relative degree of positive medicine binding ability with PD-L1; the negative spike in the green frame showed the binding ability of positive medicine (baicalin, HQG) with PD-L1.

Baicalin (HQG) has been reported to have clear PD-L1 inhibitory activity, and HQG was selected as a positive drug [44]. HQG was analyzed by the PLT system and generated two HPLC chromatograph graphs of the blank group and the experimental group of positive medicine. After deduction, the theoretical difference in these two groups (Figure 1), the relative capacity of protein binding (RCP) of HQG was 37.7% (Ap_(HQG)_ = 941,684, An_(HQG)_ = 586,887) fully testifying to the effectiveness of the new PLT system on screening small-molecule inhibitors of PD-L1.

### 2.2. PD-L1 Binding Active Compounds in TA Screened by New PLT System

Based on the literature review, there is currently no mature method for rapid inhibitor selection for PD-L1 in a complex TCM system. To explore this understudied field, a PLT strategy was used to establish a novel and rapid screening system for natural small-molecule modulators inhibiting PD-L1. In the control group and experimental group, the mixed compounds in the TA sample were both analyzed, and uncombined compounds with different columns were separated using HPLC. PD-L1 binding active compounds were retained on the protein column but not the NHS-activated agarose column without PD-L1 protein. As a result, the results of HPLC analysis, based on quantitation of corresponding compounds peak areas, were discrepant in the control and experimental groups.

**Figure 2:**
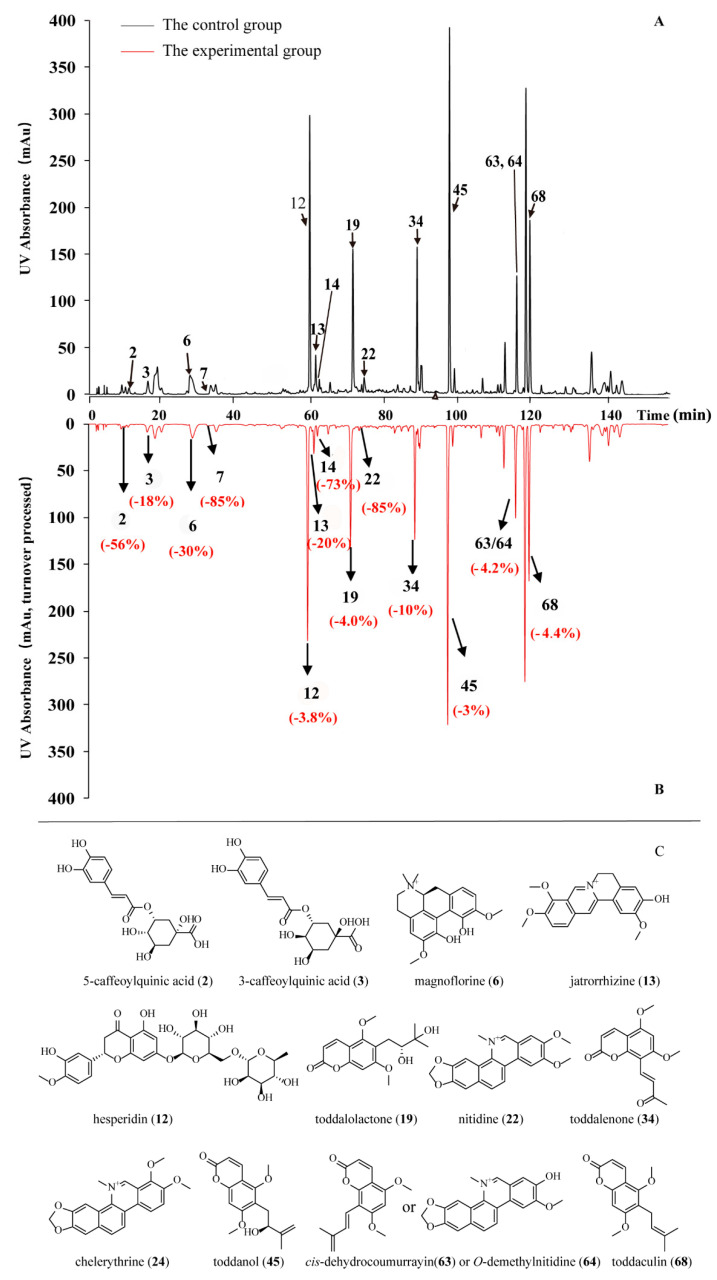
Comparison of HPLC chromatograph between the control group (**A**) and experimental group (**B**). The peaks indicated by the purple arrow are the ones showing significant peak area reduction; RCPs in the parentheses show the relative degree of compound binding ability with PD-L1 (**C**). The chemical structural formulas of 13 identified active compounds in TA show significant peak area reduction in Table 1.

As shown in Figure 2, 13 peaks (**2**, **3**, **6**, **7**, **12**, **13**, **14**, **19**, **22**, **34**, **45**, **63**/**64,** and **68)** showed significant peak area reduction, indicating PD-L1 binding activity (Table 1 and Supporting Information, Table S1).

Due to low ultraviolet absorption, it was difficult to use RCP to evaluate Peak **23**; however, a decreasing trend in the peak area could be observed. To avoid the omission of active compounds, peak **23**’s relative compound was included in the subsequent SPR verification process.

According to a mass spectrometry chromatograph and data (Supporting Information, Figure S1), 78 compounds from the TA sample were identified by their LC/MS behaviors with the assistance of standard compounds. Of these, 14 demonstrated PD-L1 binding potential: peak **2** (5-Caffeoylquinic acid, i.e., neochlorogenic acid, 5-CQA), peak **3** (3-Caffeoylquinic acid, i.e., chlorogenic acid, 3-CQA), peak **6** (magnoflorine, MLHG), peak **12** (hesperidin, CPG), peak **13** (jatrorrhizine, YGZ), peak **19** (toddalolactone, FLNZ), peak **22** (nitidine, LMZZ), peak **24** (chelerythrine, BQHZ), peak **34** (toddalenone, TONE), peak **45** (toddanol, TOOL), peak **63** or peak **64** (*cis*-dehydrocoumurrayin or *O*-demethylnitidine), and peak **68** (toddaculin, FLXS) (Table 1 and Supporting Information, Table S1).

### 2.3. SPR Validation Results: PD-L1 Binding Affinity at the Molecular Level

**Figure 3:**
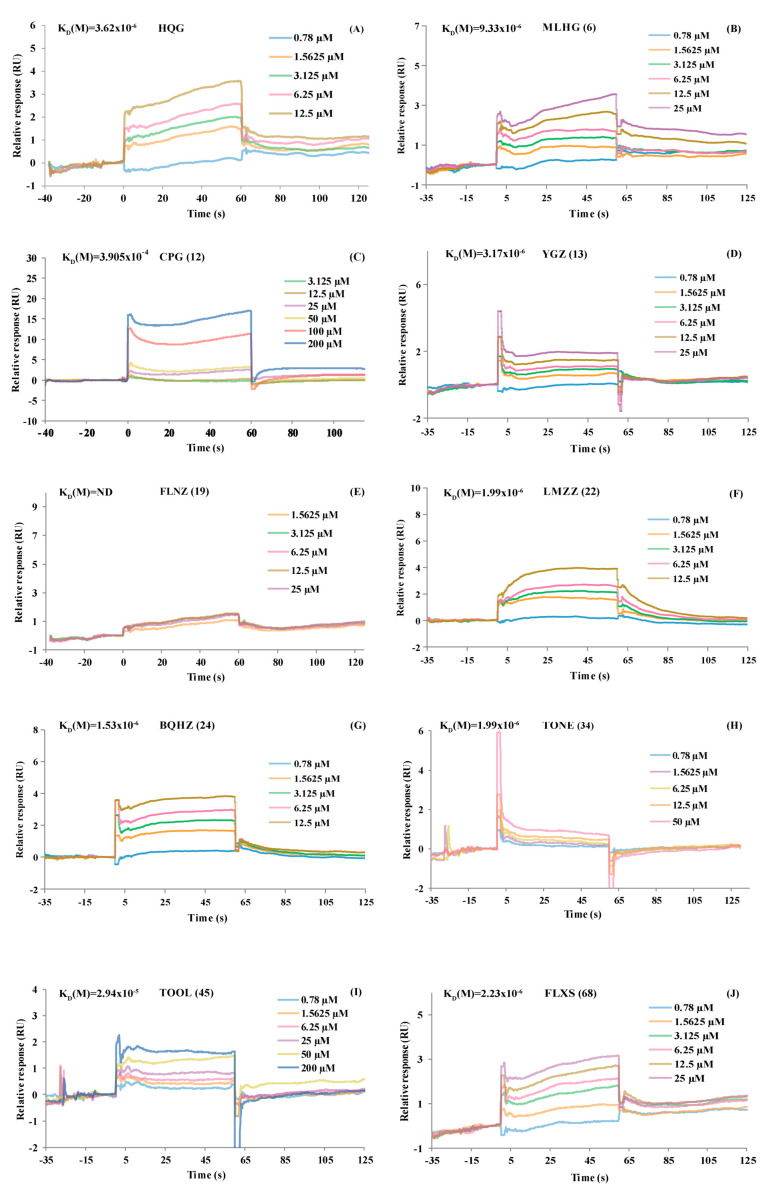
PD-L1 binding affinity validation results of the potential compounds from TA by SPR. (**A**) HQG and PD-L1, (**B**) MLHG (**6**) and PD-L1, (**C**) CPG (**12**) and PD-L1, (**D**) YGZ (**13**) and PD-L1, (**E**) FLNZ (**19**) and PD-L1, (**F**) LMZZ (**22**) and PD-L1, (**G**) BQHZ (**24**) and PD-L1, (**H**) TONE (**34**) and PD-L1, (**I**) TOOL (**45**) and PD-L1, (**J**) FLXS (**68**) and PD-L1.

Taking compound commercial availability into account, we finally selected 11 of the 14 compounds showing significant response signal reductions for subsequent studies. SPR was conducted to evaluate the PD-L1 binding affinities of these 11 compounds. 5-CQA (**2**), 3-CQA (**3**), MLHG (**6**), CPG (**12**), YGZ (**13**), FLNZ (**19**), LMZZ (**22**), BQHZ (**24**), TONE (**34**), TOOL (**45**), and FLXS (**68**). Baicalin (HQG) was chosen as a positive control. Their affinities were evaluated based on 1:1 kinetic binding and steady-state affinity models (Figure 3 and Supporting Information, Figure S2) using Biacore 8k evaluation software.

Based on the SPR results, HQG demonstrated a PD-L1 protein binding affinity (K_D_ = 1.60 × 10^−6^) (Figure 3A). According to a previous study, the PD-L1 binding affinities of 3-CQA and 5-CQA were K_D_ = 1.71 × 10^−5^ and K_D_ = 8.13 × 10^−5^, respectively [38]. As shown in Figure 3B–J, the SPR results also revealed that 10 compounds—CPG (**12**, K_D_ = 3.905 × 10^−4^), TOOL (**45**, K_D_ = 2.94 × 10^−5^), LMZZ (**22**, K_D_ = 9.46 × 10^−6^), MLHG (**6**, K_D_ = 9.33 × 10^−6^), YGZ (**13**, K_D_ = 3.17 × 10^−6^), FLXS (**68**, K_D_ = 2.23 × 10^−6^), TONE (**34**, K_D_ = 1.99 × 10^−6^), and BQHZ (**24**, K_D_ = 1.53 × 10^−6^)—had PD-L1 binding affinities, which implied positive results in the PD-L1 binding activity study. Therefore, the rapid PLT system for natural small-molecule inhibitors of PD-L1 had good reliability. Exceptionally, the negative SPR signal for FLNZ (**19**, K_D_: ND) can be accounted for by the poor RCP value (−3.95%) from the computation of the PLT system.

### 2.4. Cytopharmacology Validation Results: PD-1/PD-L1 Inhibiting Activity at the Cellular Level

#### 2.4.1. Cytotoxicity and Concentration Design of the In Vitro Study

**Figure 4:**
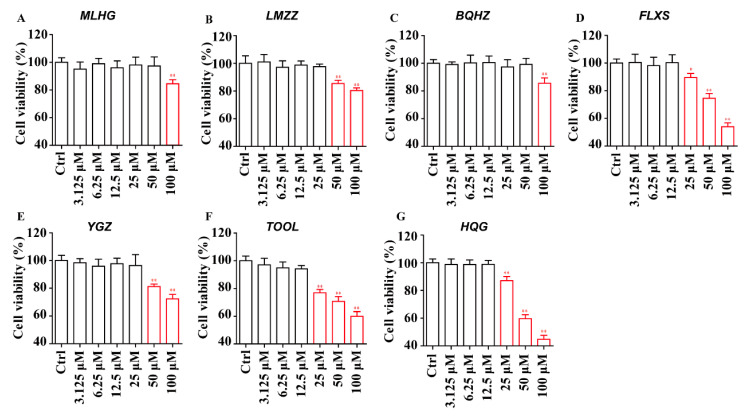
Effects of MLHG (**6**), LMZZ (**22**), BQHZ (**24**), FLXS (**68**), YGZ (**13**), TOOL (**45**), and HQG on the cell viability of A549 cells. Incubation in the presence of MLHG, **6** (**A**); LMZZ, **22** (**B**); BQHZ, **24** (**C**); FLXS, **68** (**D**); YGZ, **13** (**E**); TOOL, **45** (**F**), or HQG (**G**). At 3.125, 6.25, 12.5, 25, 50, and 100 μM, the cell viability of A549 cells was detected by the CCK8 assay, and the statistical histogram of the cell viability ratio of the indicated groups was revealed. The measurement of all results was presented with the mean along with the SD for every experimental group in gradient concentrations. (* *p* < 0.05, ** *p* < 0.01).

Based on the positive results described in Section 2.3, as well as the investigation that has not been previously reported in PD-1/PD-L1-related activity studies, six compounds—MLHG (**6**), LMZZ (**22**), BQHZ (**24**), YGZ (**13**), FLXS (**68**), and TOOL (**45**)—were further examined on cytotoxicity, which, for each condition, was performed in triplicate. HQG was chosen as the positive control. Cytotoxicity analysis of the CCK8 assay showed that FLXS (**68**), TOOL (**45**), and HQG at 25 μM, LMZZ (**22**), and YGZ (**13**) at 50 μM, and MLHG (**6**) and BQHZ (**24**) at 100 μM could significantly inhibit the viability of A549 cells (Figure 4). Therefore, to limit the influence of cytotoxicity, the following in vitro studies were carried out under diluted concentration.

#### 2.4.2. Immunofluorescence Validation Results: PD-1/PD-L1 Inhibiting Activity

For the immunofluorescence assay, an Alexa Fluor 488-labeled PD-1 Fc fragment was added to the A549 cell culture dishes and co-cultured for 24 h. All unbound PD-1 protein was washed away, and the fluorescent fragment bound to PD-1 in the A549 cells was retained and detected. Next, the fragment was co-cultured in a complete medium for 24 h in the presence of MLHG (**6**), LMZZ (**22**), BQHZ (**24**), YGZ (**13**), FLXS (**68**), and TOOL (**45**) at the same concentrations as Section 2.4.1. Then, the cell slides were pretreated, and laser scanning confocal microscopy (LSCM) was used to compare the intensity of the final fluorescence signal of these small-molecule compounds for the purpose of determining their PD-1/PD-L1 blocking ability.

**Figure 5:**
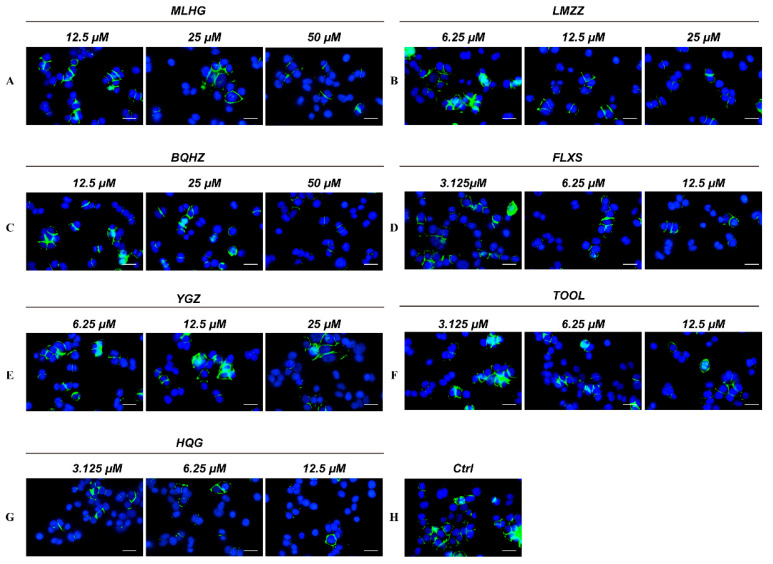
Effects of MLHG (**6**) (**A**), LMZZ (**22**) (**B**), BQHZ (**24**) (**C**), FLXS (**68**) (**D**), YGZ (**13**) (**E**), TOOL (**45**) (**F**), HQG (**G**), and solvent (**H**) on the interaction of the PD-1 Fc fragment and PD-L1 in A549 cells. After incubation in the presence of MLHG, **6** (**A**); LMZZ, **22** (**B**); BQHZ, **24** (**C**); FLXS, **68** (**D**); YGZ, **13** (**E**); TOOL, **45** (**F**), or HQG (**G**) for 24 h, LSCM images showed the association of PD-L1 on A549 cell membranes with PD-1/Fc protein. Green fluorescence indicated the conjugated PD-1 Fc fragment; the nucleus was stained by DAPI (blue fluorescence). The microscopy images of PD-1/Fc fluorescence in the presence of different compounds were presented (**A**–**G**).

Figure 5 shows fluorescence images of the six TA compounds used in the experimental group and one positive medicine and one blank used in the control group. Supporting Information, Figure S3a–g depict the statistical histograms of integral optical density of several experimental groups. The results indicated that the positive control HQG at 3.125 μM, MLHG (**6**) at 25 μM, LMZZ (**22**) at 12.5 μM, BQHZ (**24**) at 50 μM, YGZ (**13**) at 25 μM, FLXS (**68**) at 6.25 μM, and TOOL (**45**) at 6.25 μM significantly blocked the combination of the PD-1 Fc fragment with the A549 cells (*p* < 0.01). Furthermore, fluorescence intensity decreased as the target compound and positive medicine concentrations increased, indicating that the small-molecule compounds blocked the combination of PD-1 and PD-L1 in a dose-dependent fashion.

#### 2.4.3. Inhibitors May Promote A549 Apoptosis When Cocultured with PBMCs Induced by PD-1/PD-L1

**Figure 6:**
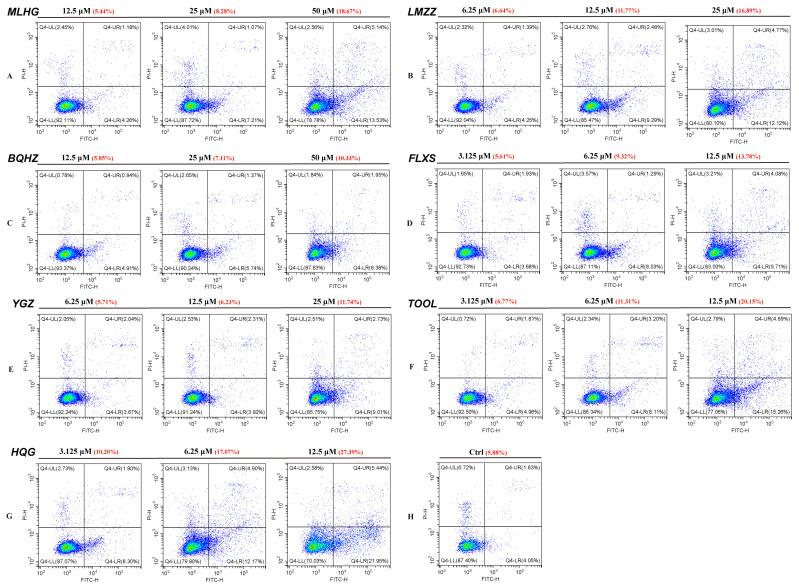
Effects of MLHG (**6**) (**A**), LMZZ (**22**) (**B**), BQHZ (**24**) (**C**), FLXS (**68**) (**D**), YGZ (**13**) (**E**), TOOL (**45**) (**F**), HQG (**G**), and solvent (**H**) on PBMCs triggering A549 cells apoptosis. Cocultivations of A549 cells and PBMCs were conducted in the presence of MLHG, **6** (**A**); LMZZ, **22** (**B**); BQHZ, **24** (**C**); FLXS, **68** (**D**); YGZ, **13** (**E**); TOOL, **45** (**F**), or HQG (**G**) for 24 h, followed by apoptosis analysis of A549 cells with FCM. The measurement of all results was presented with the mean along with the SD for individual experimental groups in relevant concentrations. Red numbers in the parentheses show the cell apoptosis rate of A549 at the corresponding concentrations of those compounds.

The PD-1/PD-L1 inhibiting effect of small molecules would eventually promote the T cell’s killing effect on tumor cells. Therefore, the effects of the six TA compounds described in Section 2.4.1 were investigated, and HQG on the auxo-action of A549 apoptosis induced by PD-1/PD-L1 when co-cultured with PBMCs for 24 h, which were carried out in the presence of A-G at the same concentrations as Section 2.4.1. Then, FCM was conducted to estimate apoptosis of the A549 cells. The results showed that HQG and (*p* < 0.01) significantly and dose-independently promoted the apoptosis of A549 cells (Figure 6). Figure S4 (Supporting Information) shows statistical histograms of the apoptosis rate. Among these six compounds, FLXS (**68**) and TOOL (**45**) showed stronger effects.

## 3. Experimental Section

### 3.1. Materials

In this study, a PD-L1 protein–ligand trapping (PLT) system for rapid screening of small molecule inhibitors in a complex TCM system was established for the first time, in the process of which, the indispensable materials, including PD-L1 DNA plasmid (extracellular fragment), PD-L1 recombinant protein expressed in mammalian cells pathway, PD-L1 protein column, TA sample solution, and positive medicine solution, were obtained in the laboratory.

#### 3.1.1. Reagents and Chemicals

Optima Liquid Chromatogram/mass spectrum (LC/MS) grade formic acid (Thermo Fisher Scientific, Beijing, China), HPLC grade acetonitrile and methanol (Duksan Pure Chemicals Co., Ltd., Ansan-si, Republic of Korea), and Sinofection transfection reagent STF02 (Sino Biological Inc., Beijing, China) were purchased for the present study. We obtained ultrapure water using the Milli-Q Water Purification System (Millipore, Bedford, MA, USA). A Series S CM5 chip, PBS 1× (a 1 mM phosphate buffer containing 2.7 mM KCl, 137 mM NaCl with 0.05% surfactant P20, and a final pH of 7.4), a coupling buffer (sodium acetate, pH 5.0), an amino coupling kit (0.4 M *N*-hydroxysuccinimide (NHS), 0.1 M 1-ethyl-3-(3-dimethylaminopropyl)-carbodiimide hydrochloride (EDC), 1.0 M ethanolamine (pH 8.5), and a regeneration reagent (50 mM NaOH)) were purchased from GE Healthcare (Uppsala, Sweden). A plasmid extraction kit was purchased from Omega Bio-Tek (Norcross, GA, USA). DH5α competent *Escherichia coli* (Solarbio Inc., Beijing, China), SMM 293-TII serum-free medium (Sino Biological Inc., China), A549 cell line (ATCC, Rockville, MD, USA), RPMI 1640 medium (GIBCO, Invitrogen Corporation, Grand Island, NY, USA), Fetal Bovine Serum (FBS, GIBCO, Invitrogen Corporation, NY, USA), streptomycin, penicillin (Sigma, St. Louis, MO, USA), and PBMCs (BIORN Life Science Co., Ltd., Beijing, China) were prepared for the *Escherichia coli* and cell culture.

Chlorogenic acid, neochlorogenic acid, magnoflorine, nitidine chloride, chelerythrine, toddaculin, jatrorrhizine, toddanol, and baicalin were purchased from Chengdu Desite Biotech Inc., Chengdu, China. The purities of these standard compounds were tested using HPLC and determined to be greater than 98%. Dry slices of TA were purchased from Yulin Drug Market, Yulin, China. We commercially acquired all other chemicals in analytical grade.

#### 3.1.2. *Escherichia coli* and Cell Culture

DH5α competent *Escherichia coli* was cultivated in Luria–Bertani (LB) broth with 50 µg/mL kanamycin at 37 °C after transformation using the heat shock method. The HEK 293F cell line (obtained from Liu Tao Laboratory, PKUHSC, Beijing, China) was suspension cultivated in SMM 293-TII serum-free medium with gentle shaking (175 rpm). The A549 cell line and PBMCs were cultivated in RPMI 1640 medium with 10% FBS, 100 µg/mL streptomycin, and 100 U/mL penicillin, as well as PBMCs with an additional 200 μg/mL PHA. All other cells were cultivated at standardly recommended culture conditions.

#### 3.1.3. TA Sample Solution Preparation and Positive Medicine Selection

The TA sample was crushed into powder and was collected after sieving through a 65-mesh sieve. Then, 2.0 g of the sieved TA powder was accurately weighed and extracted using the ultrasonic method (250 W, 33 kHz) for 30 min with 70% (*v*/*v*) aqueous ethanol (50 mL). The original sample solution was centrifuged at 10,000 rpm for five minutes after extraction. A 4% gelatin solution was dropped into the supernatant until no precipitation appeared. Then, the mixture was centrifuged at 10,000 rpm for 10 min to remove tannins and to extract the supernatant for dryness. A total of 20 mL of 70% (*v*/*v*) aqueous ethanol was added to fully dissolve the dried supernatant. The final sample solution was analyzed after being filtered with a 0.22-µm membrane.

It has been reported that baicalin can significantly suppress tumor growth and proliferation through attenuating PD-L1 upregulation [44], and in the preliminary experiment, baicalin was proved to be active in the SPR experiment binding with PD-L1; hence, baicalin was selected as a positive medicine.

#### 3.1.4. PD-L1 DNA Plasmid (Extracellular Fragment) Preparation and hPD-L1 Recombinant Protein Expression in Mammalian Cells

**Figure 7:**
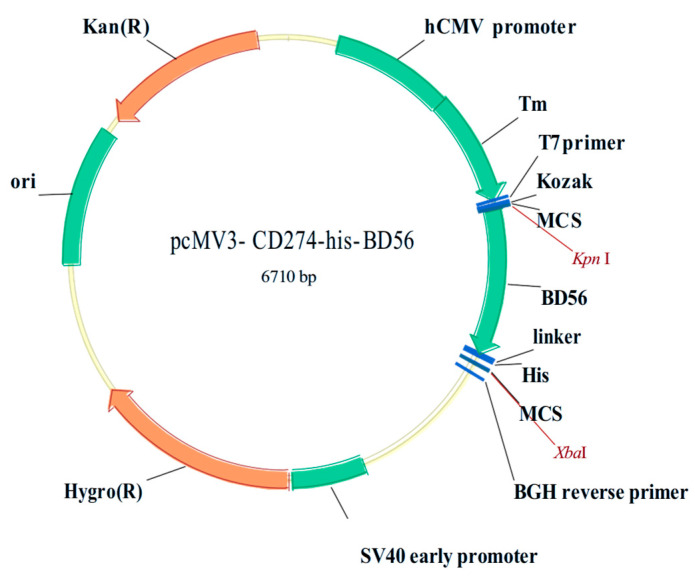
Recombinant human PD-L1 (extracellular fragment) DNA plasmid pCMV3-CD274-his-BD56.

An expression vector carrying pCMV3-CD274, a sequence encoding a fused protein, was prepared by cloning the PD-L1 sequence in an extracellular fragment (NCBI Reference Sequence: NC_000009.12) into the pCMV3 plasmid. Then, the vector was then flagged by an His-tag at the *C*-terminus for protein purification. The pCMV3-CD274-his-BD56 plasmid was commercially constructed and synthesized by Sino Biological Inc. (Figure 7).

The produced expression vector, pCMV3-CD274-his-BD56, was transformed into DH5α cells, and the positive clones were selected and proliferated. Samples containing 3 × 10^5^ HEK-239F cells were treated with an optimized mixture of target plasmid and STF02 based on the manufacturer’s instructions. Then, 48 h post-transfection, the cells were harvested and then centrifuged at 4 °C for 10 min. Next, the cell suspension was passed through a Ni-NTA column in AKTA avant (GE Healthcare, Uppsala, Sweden). The target protein, which consisted of extracellular fragments of human PD-L1 protein and an His-tag, was expressed and purified.

#### 3.1.5. PD-L1 Protein Immobilized Affinity Column (PD-L1 Protein Column) Synthesis

**Figure 8:**
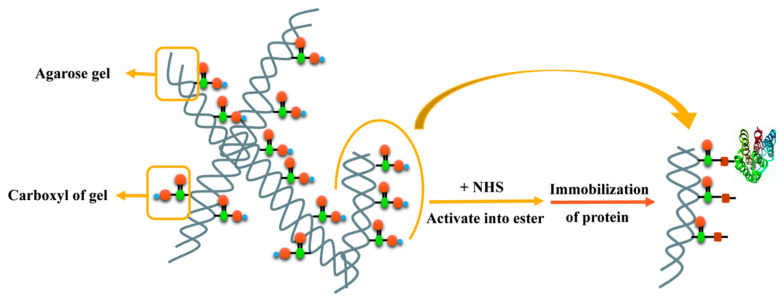
Schematic diagram of the reaction between NHS (agarose gel) and primary amino (protein). Red squares represent the -CO-NH- structures.

A novel PD-L1 protein immobilized affinity column (PD-L1 protein column) was created in order to retain the original activity of PD-L1 protein on the column for high throughput screening. Its filler matrix was NHS-activated agarose, and the PD-L1 protein was immobilized using the activated ester method (Figure 8). Given agarose’s strong nonspecific adsorption to various natural products, which caused a large deviation and false positive results in the PLT system, the concentration of NaCl surfactant of spent regenerant was optimized, and the pH level was explored by adjusting the ratio of Na_2_HPO_4_ and NaH_2_PO_4_ to reduce the nonspecific adsorption in affinity chromatography.

### 3.2. Methods

**Figure 9:**
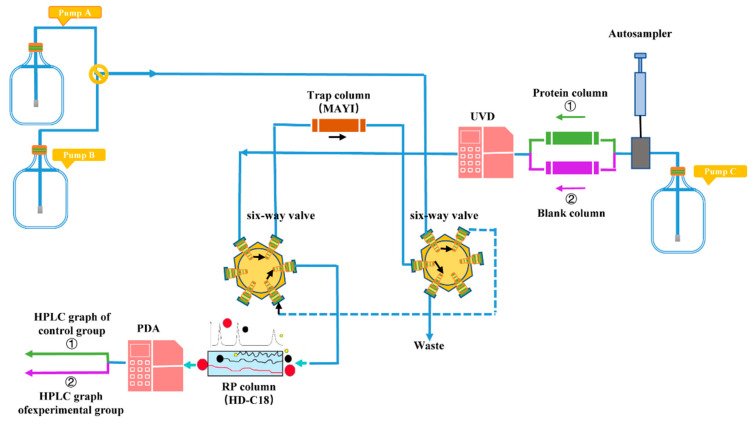
Composition of the small-molecule PD-L1 inhibitor trapping system via the PLT strategy (at position 1).

**Figure 10:**
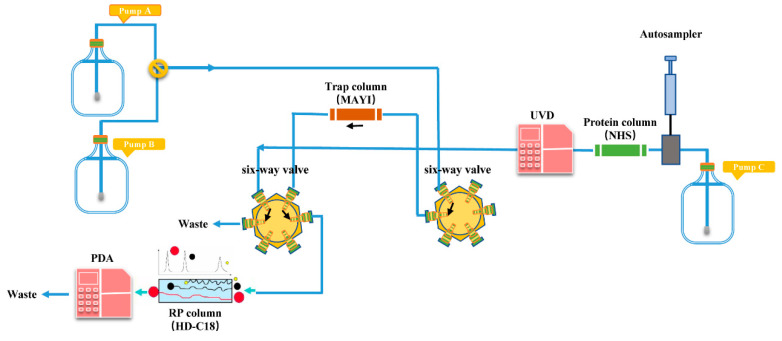
Composition of the small-molecule PD-L1 inhibitor trapping system via the PLT strategy (at position 0).

Three functional units for the composition of the PLT system were set, which were the HPLC-PDA unit, the MS unit, and the PD-L1 angling unit. The HPLC-PDA unit is the conventional HPLC analysis system, in which the analytical column part is separated into the PD-L1 angling unit. The specific content was provided in the Supporting Information. The PLT system enabled concurrent sample analysis and protein–ligand interaction monitoring through coordinated operation of dual six-port valves with valve positions 1 and 0 defined as study-specific operational states for sequential bioaffinity capture and chromatographic resolution. In the experimental group, valve position 1 directed the mobile phase through a PD-L1-functionalized angling unit for dynamic ligand-receptor interaction analysis, followed by unbound compound immobilization on a trap column prior to PDA detection (Figure 9). Subsequent valve switching to position 0 initiated a reversed-phase separation pathway, wherein trapped components underwent secondary chromatographic resolution coupled with HPLC-MS characterization (Figure 10). For controlled validation, an identical valve-switching protocol was applied to NHS-activated agarose columns devoid of PD-L1, generating baseline chromatographic profiles under equivalent analytical conditions. Differential compound identification was achieved through comparative quantification of peak area discrepancies between experimental and control chromatograms, statistically validated to confirm PD-L1-specific binding interactions. The differential PD-L1-binding compounds were identified by analyzing significant differences in peak areas through a comparative assessment of the two graphs mentioned above.

The protein-binding capacity of these compounds was evaluated using the relative capacity of protein binding (RCP), where An represents the peak area of the compound flowing through the blank column and Ap represents the peak area of the corresponding compound flowing through the protein column. The formula for RCP is as follows:

$$RCP=\frac{A_{p}-A_{n}}{A_{n}}\times 100\%$$

After the screening, SPR and cytopharmacology were used to validate the activity and obtain rich information on potential PD-L1 inhibitors from natural compounds. The specific methods are provided in the Supporting Information.

## 4. Discussion

This study pioneers a bioaffinity-integrated chromatographic trapping (PLT) platform for rapid discovery of PD-L1-targeting small molecules from TA extracts, demonstrating its efficacy through multi-tiered validation. Six of the compounds were shown to suppress PD-1 and PD-L1 in a significant, dose-dependent fashion. Thus, the established PLT system was proven to be effective and efficient. This work blazed a new trail to the efficient exploration of small-molecule PD-L1 inhibitors and provided a wealth of information on lead compounds for drug design according to the PD-L1 active domain. Moreover, this approach could promote the drug design process targeting PD-1/PD-L1 up to other checkpoints for oncotherapy.

As for NHS-activated agarose gel, it is a biological affinity chromatographic separation medium formed by agarose gel microspheres activated by N-hydroxysuccinimide sodium salt. Methodologically, the covalent immobilization of conformationally intact PD-L1 onto NHS-activated agarose via N-hydroxysuccinimide ester chemistry provided a stable bioaffinity interface. The method for immobilizing the protein, however, cannot fully immobilize the incubated protein on the gel column, which makes it difficult to quantify the amount of immobilized protein. Not all proteins can be fixed to the packing, depending on whether the protein is purified and expresses a label that can bind to the packing. It may be possible to continue to experiment with different fillers and proteins to expand the scope of application of the screening system.

The MAYI column acts as an affinity post-treatment module in the PLT system, trapping unbound small molecules eluted by the PD-L1 column by hydrophobic retention while filtering displaced protein aggregates by size exclusion. However, in screening, we found that the column had limited retention ability for small fractions with strong fat solubility, which led to a certain selectivity for screening medicinal materials. In view of this phenomenon, it may be feasible to change the MAYI column type in the screening process of different medicinal materials to increase the scope of application to the screening of medicinal materials. In addition to the above optimization of the protein column and MAYI column, after the establishment of this screening system, it can not only expand the screening of PD-L1 inhibitors in different medicinal materials but also try to screen other protein targets of natural product inhibitors in Chinese medicine and increase the universality of the system.

It is worth noting that, in addition to inhibiting PD-L1, the active inhibitors identified above have also been reported to exert anticancer effects through other mechanisms of action in related studies [45,46,47,48,49]. Given the close association between PD-L1 and cancer development, it remains to be further explored whether the anticancer effects of these compounds are directly linked to PD-L1 inhibition or involve synergistic interactions with other pathways.

## 5. Conclusions

This study established a novel protein–ligand trapping system integrating bioaffinity chromatography with orthogonal analytical detection to rapidly identify programmed death ligand 1 inhibitors from either single compounds, mixtures, or complex natural extracts. Applied to *Toddalia asiatica* (L.) Lam extracts, the system identified 14 compounds with programmed death ligand 1 binding potential, 11 of which exhibited specific programmed death ligand 1 binding affinity in surface plasmon resonance assays. Cytopharmacological evaluations confirmed dose-dependent programmed cell death protein-1/programmed death ligand 1 blockade through immunofluorescence quantification of receptor-ligand interactions, complemented by cell counting kit-8 cytotoxicity assays to establish non-toxic working concentrations. Functional validation in a peripheral blood mononuclear cell (PBMC)-human lung cancer cell line (A549) co-culture model demonstrated significant enhancement of apoptosis induction through flow cytometric quantification, mechanistically linked to programmed death ligand 1 signaling pathway disruption. including benzophenanthridine alkaloids (nitidine, chelerythrine), an aporphine alkaloid (magnoflorine), a protoberberine alkaloid (jatrorrhizine), a coumarin (toddaculin), and a sesquiterpenoid (toddanol)—were validated as programmed death ligand 1 binders, demonstrating the inhibitory potential of alkaloid and coumarin scaffolds for programmed cell death protein-1/programmed death ligand 1 blockade. The methodological innovation of this study lies in the novel preparation of programmed death ligand 1 eukaryotic protein with complete biological functions used in the programmed death ligand 1 angling unit for inhibitor screening as well as repurposing conventional instrumentation for multidimensional bioactivity analysis. By integrating the protein–ligand trapping platform with molecular binding kinetics, cellular profiling, and phenotypic validation, this work establishes a translatable framework for discovering immune checkpoint inhibitors from natural products.

## Figures and Tables

**Table 1:** Compounds in TA that showed significant peak area reduction or reducing trend.

NO.	Peak	TR (min)	An	Ap	RCP (%)	Peak Area Drop Value
**2**	7	10.278	217,068	95,476	−56.015626	121,592
**3**	8	15.546	497,316	405,786	−18.404797	91,530
**6**	11	26.808	1,489,829	1,042,284	−30.040025	447,545
**7**	12	32.328	395,747	58,265	−85.27721	337,482
**12**	15	58.773	5,312,326	5,110,540	−3.7984491	201,786
**13**	16	60.376	818,092	656,451	−19.758291	161,641
**14**	17	61.762	108,244	29,147	−73.072872	79,097
**19**	20	70.308	3,480,298	3,342,819	−3.9502077	137,479
**22**	22	73.297	425,276	62,145	−85.387137	363,131
**34**	27	87.459	2,469,279	2,220,585	−10.071523	248,694
**45**	29	96.103	6,282,474	6,069,301	−3.3931378	213,173
**63**/**64**	39	114.078	2,164,023	2,073,044	−4.2041605	90,979
**68**	41	116.525	5,727,162	5,472,947	−4.4387604	254,215
**24**	23	76.127	-	-	-	-

## Data Availability

The data presented in this study are available on request from the corresponding author.

## Supplementary Materials

The following supporting information can be downloaded at https://www.mdpi.com/article/10.3390/molecules30081754/s1: Figures S1–S4; Table S1. Reference [50] is cited in the supplementary materials.
