# Supplementary material for: A New Protein–Ligand Trapping System to Rapidly Screen and Discover Small-Molecule Inhibitors of PD-L1 from Natural Products
Source: Molecules. 2025 Apr 14;30(8):1754. doi: 10.3390/molecules30081754 (PMC12029895; doi:10.3390/molecules30081754)
Supplement: Supplementary file 1 [file molecules-30-01754-s001.zip › molecules-3546698-supplementary.pdf]

# Supporting Information

## A New Protein-Ligand Trapping System to Rapidly Screen and Discover Small-Molecule Inhibitors of PD-L1 from Natural Products

Yazhuo Huang [a,1], Senfeng Sun[a,1], Runxin Yin [a], Zongtao Lin[c], Daidong Wang[a], Wanwan Wang[a], Xiangyu Fu[a], Jing Wang[a], Xinyu Lei[a], Mimi Sun[b], Shizhong Chen[a]*, Hong Wang [a*, #1]

[a]*School of Pharmaceutical Sciences, Peking University, Beijing 100191, China.*
[b]*School of Pharmacy, Shaanxi University of Chinese Medicine, Xianyang 712046, China.*
[c]*Department of Chemistry, University of Pennsylvania, Philadelphia, PA 19104, United States.*

*Corresponding authors. E-mail address: chenshizhong66@163.com (SZ.C.); hw9505@bjmu.edu.cn (H.W.)
[1] This is the first corresponding author.

Table of Contents

Experimental Section ........ S3

1. Methods ........ S3

1.1 Establishment and validation of the small-molecule PD-L1 inhibitor trapping system via PLT strategy ........ S3

1.1.1 Apparatus, components, and operational mode ........ S3

1.1.2 Validation of the PLT system through positive medicine ........ S3

1.1.3 HPLC and ESI-IT-TOF (Q-TOF)-MS conditions ........ S3

1.2 Activity validation using SPR ........ S3

1.3 Activity validation using cytopharmacology ........ S3

1.3.1 Cytotoxicity ........ S4

1.3.2 Direct inhibition of PD-1 and PD-L1 interactions ........ S4

1.3.3 Auxo-action on A549 apoptosis ........ S4

**Figure S1.** HPLC fingerprint chromatograph and total ion chromatograph of IT-TOF (A) and Q-TOF (B) of TA. ........ S5

**Table S1.** Characterization of 78 compounds in TA in the positive ion mode of Q-TOF-$MS^2$ S6

**Figure S2.** PD-L1 binding activity validation results in affinity models of the potential compounds from TA by SPR ........ S14

**Figure S3.** Effects of MLHG (a), LMZZ (b), BQHZ (c), FLXS (d), YGZ (e), TOOL (f), and HQG (g) on the interaction of PD-1 Fc and in PD-L1 A549 cells ........ S15

**Figure S4.** The statistical histogram of apoptosis rate of MLHG (a), LMZZ (b), BQHZ (c), FLXS (d), YGZ (e), TOOL (f), HQG (g) ........ S16

## Experimental Section

1. Methods

### 1.1 Establishment and validation of the small-molecule PD-L1 inhibitor trapping system via PLT strategy

#### 1.1.1 Apparatus, components, and operational mode

The PLT system consisted of three functional units. First, the HPLC-PDA unit was used for chromatographic analysis and equipped with one CBM-20A system controller, two LC-20ADXR pumps, two LC-20AD pumps, one DGU-20A5 degasser, one SIL-20ACXR autosampler, one CTO-20AC column oven, one trap column (Kromasil 100-5-C8, 5 µm, 4.6×50 mm), one reverse phase analytical chromatographic column (RP column) (Shim-pack Scepter HD-C18-80, 5 µm, 4.6×250 mm), one PDA-M20A DAD detector, one PDA-20A UV detector, two six-port valves, and LabSolutions WorkStation software. Second, the MS unit consisted of an IT-TOF(Q-TOF)-MS, which was equipped with an ESI interface for mass spectrometry. These two units constituted the analytic module. Third, the PD-L1 angling unit consisted of a PD-L1 protein column or an NHS-activated agarose column without PD-L1 protein; this unit constituted the angling module.

#### 1.1.2 Validation of the PLT system through positive medicine

To validate the effectiveness of the PLT system, we took positive medicine (HQG) as a sample to evaluate the PD-L1 combining capacity, compared the HPLC chromatograph of the blank group and the experimental group of positive medicine, and analyzed the theoretical difference between these two groups.

#### 1.1.3 HPLC and ESI-IT-TOF(Q-TOF)-MS conditions

A Shim-pack Scepter HD-C18-80 column was used for separation at 30 °C in a flow velocity of 1.0 mL/min. A mobile phase in different proportions of A (water with 0.1% FA) and B (acetonitrile) was used for chromatographic separation. The linear gradient was: 0-10 min, 8→10% B; 10-25 min, 10→12% B; 25-65 min, 12→25% B; 65-95 min, 25→50% B; 95-115 min, 50→65% B; 115-145 min, 65→80% B; and 145-155 min, 80→100% B. The PDA was set at 294 nm.

The conditions of IT-TOF-MS: the gas of collision-induced dissociation (CID) was high-purity argon, the temperature was set at 200 °C, the nitrogen flow rate was 1.5 L/min. In positive mode, the spray voltage is -3.5 kV, the accelerating voltage is 3.5 kV, the detector voltage is 1.7 kV, and the collision energy is 50%. Mass correction used sodium trifluoroacetate (2.5 mmol/L) in the range of 50-1000 Da. In positive ion mode, tertiary mass spectrometry fragments were collected. Data analysis was conducted through the LCMS solution 3.8 and Formula Predictor software.

The conditions of Q-TOF-MS: SCIEX Exion LC AD System (UPLC) were used for the chromatography model. The SCIEX Triple TOF 6600$^{+}$ system was used for the mass spectrometry model. In positive ion mode, ion-source gas 1 (GS1) was set at 60 psi, ion-source gas 2 (GS2): 60 psi, curtain gas (CUR): 35 psi, ion-source temperature (TEM): 600°C, ion spray voltage (IS): 5500 V, decluster voltage (VDP): 80/-80 V, and collision energy (CE): 40±20 V. The molecular weights and retention times of different substances were qualitatively analyzed using SCIEX OS software and the PeakView matching database.

### 1.2 Activity validation using SPR

SPR experiments were performed to verify the direct suppressive function of the potential inhibiting compounds using the Biacore 8k system. The purified PD-L1 protein from **Section 1.1.4** was diluted to 25 µg/mL using sodium acetate at a pH of 5.0 for covalent immobilization on a CM5 sensor chip. The coupling amount of the hPD-L1 protein was calculated to be 13000 RU. The representative active compounds in TA were dissolved and diluted to a gradient concentration in the scope of 0.78 µM to 200 µM in a final 5% solution of dimethylsulfoxide (DMSO) PBS-P. Corrections for system errors were conducted by blank solutions without analyte, and a blank reference was conducted by a flow cell without coupling proteins. DMSO corrections were performed using a small-molecule protein assay.

### 1.3 Activity validation using cytopharmacology

The potential inhibitors of PD-L1 were further validated using cytopharmacology. As part of the validation process, disturbances to the immediate interaction between PD-1 and PD-L1 were examined.

As another part, the activation of immune cells triggering apoptosis of tumor cells through suppression of PD-1/PD-L1 was also explored.

### 1.3.1 Cytotoxicity

To confirm the safe concentrations of the selected drugs, various concentrations of the positive drug and target compounds (i.e., 3.125 μM, 6.25 μM, 12.5 μM, 25 μM, 50 μM, 100 μM) were incubated separately with A549 cells for 24 h, and a blank control group was incubated as well. We evaluated the sensitivity of A549 cells to the compounds mentioned above in gradient concentrations in the same manner as the CCK8 assay[2].

### 1.3.2 Direct inhibition of PD-1 and PD-L1 interactions

A covered glass (22×22 mm) was sunk in 75% ethanol for 2 h. After the ethanol evaporated, the covered glass was placed in a six-well plate. A549 cells in the logarithmic growth phase were seeded at $1\times10^6$ cells/well in the six-well plate and cultured at 37 °C and 5% $CO_2$. When the cells proliferated to 50%, they were starved without serum for 2 h. Then, they were co-cultured with an Alexa Fluor 488 labeled PD-1 Fc fragment in the presence of target compounds, which were selected based on their positive SPR results, in a complete medium for 24 h. The cell slides were prepared using a cytocentrifuge slide machine and then taken out, immersed in 95% ethanol for fixation, washed three times with PBS, placed on a glass slide for 5 min each time, and mounted with 4',6-diamidino-2-phenylindole (DAPI). They were observed under a laser scanning confocal microscope (LSCM), and pictures were recorded and analyzed through Image J to evaluate the average intensity of green fluorescence, which served as an index for the immediate suppression of PD-1/PD-L1 interaction.

The measurement of all data was presented with the mean along with the standard deviation (SD); we analyzed the statistical differences through a one-way "ANOVA" and Tukey's test. $P < 0.05$ was characterized as a statistically significant difference.

### 1.3.3 Auxo-action on A549 apoptosis

An FCM test was designed to measure the activation of immune cells, such as PBMCs, which trigger A549 cell apoptosis through suppression of the PD-1/PD-L1 signal. A549 cells were cultured in the logarithmic growth phase until they were 50% confluent. The separately cultured PBMC cells were resuspended in a complete medium with seven gradient concentrations and added to the six-well plate by incubating with A549 cells for 24 h. After incubation, the A549 cells were distributed in a flow tube based on the grouping situation. After twice washing with PBS, 0.5 mL of staining buffer was dispersed to each tube to resuspend the cells. Then, 5 μL of fluorescein isothiocyanate isomer (FITC) staining solution was added and mixed. After incubating in the dark for 15 min, we further mixed 5 μL of propidium iodide (PI) staining solution with each tube up. After co-incubation for 5 min, on-device detection was performed.

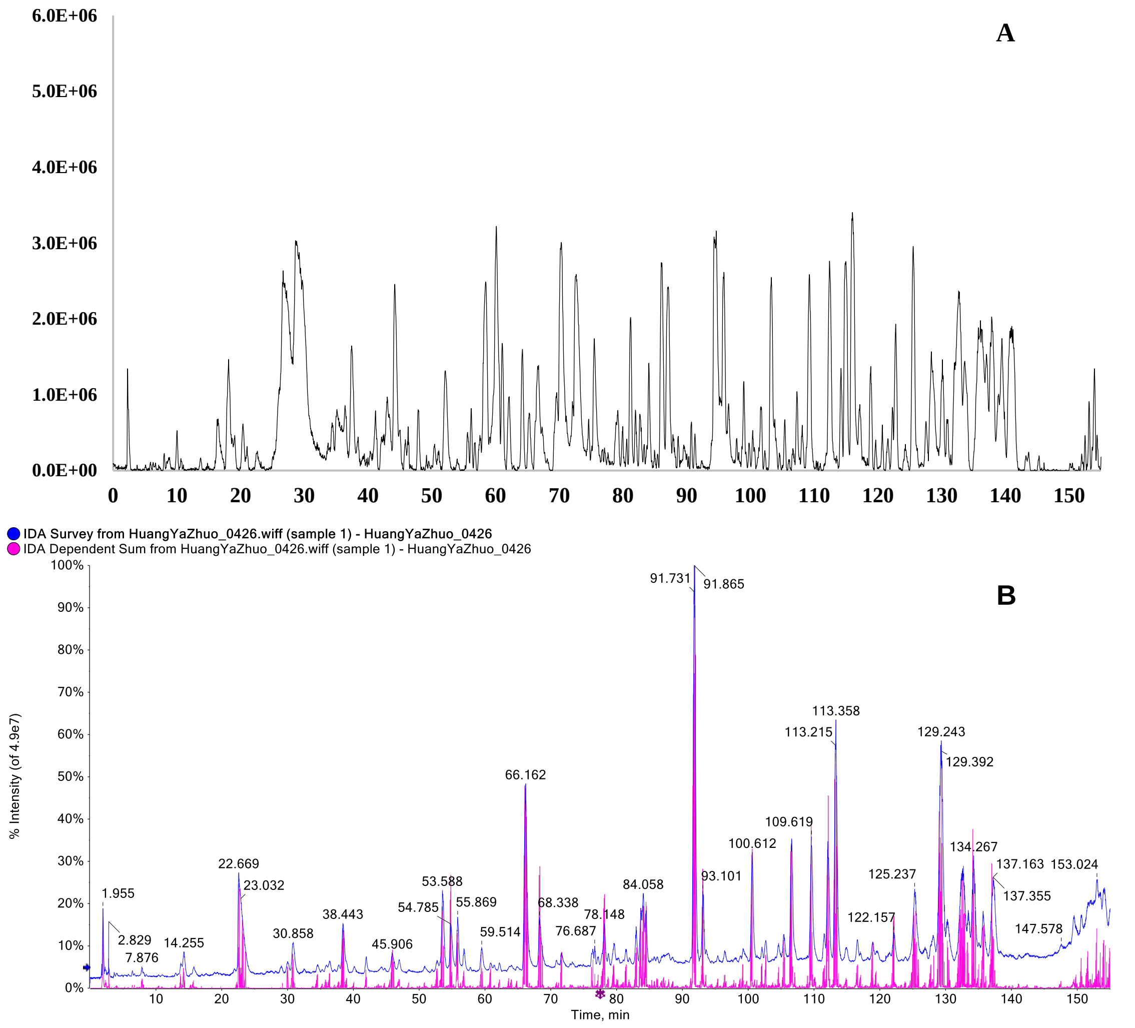


**Figure S1.** HPLC fingerprint chromatograph and total ion chromatograph of IT-TOF (A) and Q-TOF (B) of TA.

**Table S1.** Characterization of 78 compounds in TA in the positive ion mode of Q-TOF-MS[2]

| Peak | Formula | (+) ESI-MS$^n$ MS$^n$(Precursor) | Fragment ions | Identification |
|---|---|---|---|---|
| 1 | $C_6H_{11}NO_3$ | 146.1[M+H]$^+$ | 118.0864[M+H-CO]$^+$ | 4-hydroxy-n-methylproline |
| | | | 100.0754[M+H-CO-$H_2O$]$^+$ | |
| | | | 82.0650[M+H-CO-2$H_2O$]$^+$ | |
| 2* | $C_{16}H_{18}O_9$ | 355.1[M+H]$^+$ | 163.0390[M+H-QA]$^+$ | 5-caffeoylquinic acid |
| | | | 145.0279[M+H-QA-$H_2O$]$^+$ | |
| | | | 135.0440[M+H-$C_7H_{12}O_5$-$CO_2$]$^+$ | |
| | | | 117.0331[M+H-$C_9H_6O_3$-$H_2O$]$^+$ | |
| 3* | $C_{16}H_{18}O_9$ | 355.1[M+H]$^+$ | 163.0393[M+H-QA]$^+$ | 3-caffeoylquinic acid |
| | | | 145.0284[M+H-QA-$H_2O$]$^+$ | |
| | | | 135.0438[M+H-$C_7H_{12}O_5$-$CO_2$]$^+$ | |
| | | | 117.0327[M+H-$C_9H_6O_3$-$H_2O$]$^+$ | |
| 4 | $C_{18}H_{19}NO_4$ | 314.2[M+H]$^+$ | 298.1101[M+H-$CH_4$]$^+$ | N-*cis*-feruloyl tyramide |
| | | | 283.0851[M+H-$OCH_3$]$^+$ | |
| 5 | $C_{12}H_{12}O_5$ | 237.1[M+H]$^+$ | 222.0617[M+H-$CH_3$]$^+$ | 5,7,8-trimethoxycoumarin |
| | | | 191.0844[M+H-2$CH_3$-$CH_4$]$^+$ | |
| | | | 194.0699[M+H-$CH_3$-CO]$^+$ | |
| | | | 165.0710[M+H-$CH_3$-CO-$CH_2$-$CH_3$]$^+$ | |
| 6* | $C_{20}H_{24}NO_4$ | 342.2[M+H]$^+$ | 297.1145[M+H-NH$(CH_3)_2$-H]$^+$ | magnoflorine |
| | | | 282.0902[M+H-NH$(CH_3)_2$-H-$CH_3$]$^+$ | |
| | | | 265.0877[M+H-NH$(CH_3)_2$-H-$CH_3$-OH]$^+$ | |
| | | | 237.0928[M+H-NH$(CH_3)_2$-H-$CH_3$-OH-CO]$^+$ | |
| | | | 222.0685[M+H-NH$(CH_3)_2$-H-2$CH_3$-OH-CO]$^+$ | |
| 7* | $C_{20}H_{24}NO_4^+$ | 342.2[M+H]$^+$ | 297.1135[M+H-$CH_3$-CO]$^+$ | unkonw |
| | | | 266.0770[M+H-$CH_3$-CO-2$CH_3$]$^+$ | |
| 8 | $C_{10}H_8O_3$ | 177.2[M+H]$^+$ | 149.0222[M+H-CO]$^+$ | 7-methoxy-2H-1-benzopyran-2-one |
| 9 | $C_{17}H_{22}O_6$ | 323.1[M+H]$^+$ | 221.0443[M+H-CO-$C_4H_{10}$O]$^+$ | (+)-6-(2-hydroxy-3-methoxy-3-methylbutyl)-5,7-dimethoxycoumarin |
| | | | 191.0336[M+H-CO-$C_4H_{11}$O-O]$^+$ | |
| | | | 163.0417[M+H-CO-$C_4H_{12}$O-O-CO]$^+$ | |
| 10 | $C_{30}H_{26}O_{12}$ | 579.1[M+H]$^+$ | 287.0531[M+H-$C_{15}H_{14}O_6$-H]$^+$ | procyanidine B2 |
| | | | 409.0943[M+H-$C_{15}H_{14}O_6$-H]$^+$ | |
| | | | 427.1013[M+H-$C_{15}H_{14}O_6$-H-$H_2O$]$^+$ | |
| | | | 139.04[M+H-$C_{15}H_{14}O_6$-H-$H_2O$-$C_{15}H_{10}O_5$]$^+$ | |
| 11 | $C_{20}H_{24}O_4$ | 329.2[M+H]$^+$ | 151.0389[M+H-$C_{10}H_{16}$-CO-$CH_2$]$^+$ | 7-geranyloxy-5-methoxycoumarin |
| | | | 121.0640[M+H-$C_{10}H_{16}$-2CO-$CH_3$]$^+$ | |

| Peak | Formula | (+) ESI-MS^n MS^n(Precursor) | Fragment ions | Identification |
|---|---|---|---|---|
| 12* | $C_{28}H_{34}O_{15}$ | 611.3[M+H]$^+$ | 303.0884[M+H-rha-Glc]$^+$ | hesperidin |
| | | | 195.0294[M+H-rha-Glc-$C_6H_4O_2$]$^+$ | |
| | | | 153.0181[M+H-rha-Glc-$C_9H_{10}O_2$]$^+$ | |
| 13* | $C_{20}H_{20}O_4N^+$ | 339.2 [M+H]$^+$ | 323.1180[M+H-$CH_3$]$^+$ | jatrorrhizine |
| | | | 294.1146[M+H-$CH_4$-CO]$^+$ | |
| | | | 280.0988[M+H-$CH_3$-CO-$CH_3$]$^+$ | |
| | | | 265.0752[M+H-$CH_3$-CO-2$CH_3$]$^+$ | |
| 14* | $C_{22}H_{24}O_{11}$ | 465.1[M+H]$^+$ | 303.0893[M+H-Glc]$^+$ | hesperetin-7-*O*-β-d-glucopyranoside |
| | | | 177.0543[M+H-Glc-$C_6H_4O_2$]$^+$ | |
| | | | 153.0183[M+H-Glc-$C_9H_{10}O_2$]$^+$ | |
| 15 | $C_{21}H_{26}O_{10}$ | 439.2[M+H]$^+$ | 277.1085[M+H-Glc]$^+$ | sec-*O*-glucosylhamaudol |
| | | | 259.0979[M+H-Glc-$H_2O$]$^+$ | |
| | | | 217.0979[M+H-Glc-$H_2O$-CO-$CH_2$]$^+$ | |
| | | | 205.0502[M+H-Glc-$C_4H_8O$]$^+$ | |
| | | | 189.0551[M+H-Glc-$H_2O$-2CO-$CH_2$]$^+$ | |
| 16 | $C_{20}H_{15}NO_4$ | 334.1[M+H]$^+$ | 319.0853[M+H-$CH_3$]$^+$ | norchelerythrine |
| | | | 304.0611[M+H-2$CH_3$]$^+$ | |
| | | | 276.0660[M+H-2$CH_3$-CO]$^+$ | |
| 17 | $C_{11}H_{10}O_4$ | 207.1[M+H]$^+$ | 191.0022[M+H-$CH_4$]$^+$ | 5,7-dimethoxy-2H-1-benzopyran-2-one |
| | | | 164.0457[M+H-$CH_4$-CO-$CH_3$]$^+$ | |
| 18 | $C_{21}H_{22}NO_4^+$ | 352.2[M+H]$^+$ | 337.1335[M+H-$CH_3$]$^+$ | palmatine |
| | | | 336.1257[M+H-$CH_4$]$^+$ | |
| | | | 322.1093[M+H-$CH_4$-$CH_3$]$^+$ | |
| | | | 320.1093[M+H-$CH_4$]$^+$ | |
| | | | 308.1300[M+H-$CH_4$-CO]$^+$ | |
| | | | 304.0992[M+H-$CH_4$-O]$^+$ | |
| 19* | $C_{16}H_{20}O_6$ | 309.1[M+H]$^+$ | 291.1229[M+H-$H_2O$]$^+$ | toddalolactone |
| | | | 277.1075[M+H-$H_2O$-$CH_2$]$^+$ | |
| | | | 235.0601[M+H-CO-2$CH_2$-$H_2O$]$^+$ | |
| | | | 205.0501[M+H-2$CH_3$-2OH-$C_3H_4$]$^+$ | |
| 20 | $C_{21}H_{19}NO_4$ | 350.2[M+H]$^+$ | 335.1063[M+H-$CH_3$]$^+$ | dihydrochelerythrine |
| | | | 334.1320[M+H-$CH_4$]$^+$ | |
| | | | 320.0804[M+H$CH_2$-$CH_4$]$^+$ | |
| | | | 306.1076[M+H-CO-$CH_4$]$^+$ | |
| | | | 292.0876[M+H-$CH_4$-CO-$CH_2$]$^+$ | |
| 21 | $C_{12}H_9NO_3$ | 216.1[M+H]$^+$ | 188.0447[M+H-CO]$^+$ | robustine |
| | | | 173.0237[M+H-CO-$CH_3$]$^+$ | |

| Peak | Formula | (+) ESI-MS$^n$ MS$^n$(Precursor) | Fragment ions | Identification |
|---|---|---|---|---|
| | | | 145.0319[M+H-2CO-CH$_3$]$^+$ | |
| | | | 89.0355[M+H-3CO-CH$_3$-CH$_2$-NH]$^+$ | |
| 22* | $C_{21}H_{18}NO_4^+$ | 348.1[M+H]$^+$ | 332.0932[M+H-CH$_4$]$^+$ | nitidine |
| | | | 318.0772[M+H-2CH$_3$]$^+$ | |
| | | | 304.0973[M+H-CH$_4$-CO]$^+$ | |
| | | | 290.0826[M+H-2CH$_3$-CO]$^+$ | |
| | | | 275.0940[M+H-2CH$_3$-CO-CH$_3$]$^+$ | |
| 23 | $C_{11}H_{11}NO_2$ | 190.1[M+H]$^+$ | 175.0632[M+H-CH$_3$]$^+$ | N-methyl-4-methoxyquinolin-2(1H)-one |
| | | | 147.0679[M+H-CH$_3$-CO]$^+$ | |
| | | | 118.0646[M+H-CH$_3$-CO-NHCH$_3$]$^+$ | |
| 24* | $C_{21}H_{18}NO_4^+$ | 348.1[M+H]$^+$ | 332.0932[M+H-CH$_4$]$^+$ | chelerythrine |
| | | | 318.0777[M+H-2CH$_3$]$^+$ | |
| | | | 304.0980[M+H-CH$_4$-CO]$^+$ | |
| | | | 290.0824[M+H-2CH$_3$-CO]$^+$ | |
| 25 | $C_{32}H_{36}O_{14}$ | 645.22[M+H]$^+$ | 291.1248[M+H-H$_2$O-C$_{16}$H$_{16}$O$_8$]$^+$ | 3”’-*O*-demethyltoddalin |
| | | | 219.0666[M+H-2CH$_3$-C$_{16}$H$_{16}$O$_8$-C$_3$H$_8$O]$^+$ | |
| 26 | $C_{15}H_{18}O_5$ | 279.1[M+H]$^+$ | 247.0999[M+H-H$_2$O-CH$_2$]$^+$ | ulopterol |
| | | | 219.0665[M+H-H$_2$O-CH$_2$-CO]$^+$ | |
| | | | 217.0505[M+H-H$_2$O-CO$_2$]$^+$ | |
| | | | 205.0508[M+H-H$_2$O-2CO]$^+$ | |
| 27 | $C_{33}H_{38}O_{14}$ | 659.2[M+H]$^+$ | 219.0652[M+H-C$_{17}$H$_{20}$O$_9$-C$_3$H$_6$O-CH$_2$]$^+$ | toddalin A |
| | | | 145.0275[M+H-C$_{17}$H$_{20}$O$_9$-C$_3$H$_6$O-CH$_2$-OCH$_3$-CO$_2$]$^+$ | |
| | | | 177.0546[M+H--C$_{23}$H$_{30}$O$_{11}$]$^+$ | |
| 28 | $C_{15}H_{16}O_5$ | 277.1[M+H]$^+$ | 217.0499[M+H-H$_2$O-CO-CH$_2$]$^+$ | toddanin |
| | | | 161.0594[M+H-H$_2$O-3CO-CH$_2$]$^+$ | |
| 29 | $C_{14}H_{13}NO_4$ | 260.1[M+H]$^+$ | 230.0452[M+H-2CH$_3$]$^+$ | skimmianine |
| | | | 245.0694[M+H-CH$_3$]$^+$ | |
| | | | 227.0585[M+H-CH$_3$-H$_2$O]$^+$ | |
| | | | 216.0660[M+H-CH$_3$-H-CO]$^+$ | |
| | | | 199.0630[M+H-2CH$_3$-OCH$_3$]$^+$ | |
| 30 | $C_{17}H_{22}O_6$ | 323.2[M+H]$^+$ | 219.0659[M+H-CH$_3$-CH$_2$-CO$_2$-OCH$_3$]$^+$ | omphalocarpin |
| | | | 188.0472[M+H-C$_5$H$_{10}$O$_2$-2CH$_3$]$^+$ | |
| | | | 161.0596[M+H-C$_5$H$_{10}$O$_2$-2CH$_3$-CO]$^+$ | |
| 31 | $C_{15}H_{16}O_3$ | 245.1[M+H]$^+$ | 202.0644[M+H-CO-CH$_3$]$^+$ | suberosin |
| | | | 187.0400[M+H-CO-CH$_2$-OH]$^+$ | |
| 32 | $C_{15}H_{14}O_4$ | 259.1[M+H]$^+$ | 229.0499[M+H-2CH$_3$]$^+$ | luvangetin |
| | | | 201.0556[M+H-2CH$_3$-CH$_2$-CO$_2$]$^+$ | |

| Peak | Formula | (+) ESI-MS$^n$ MS$^n$(Precursor) | (+) ESI-MS$^n$ Fragment ions | Identification |
|---|---|---|---|---|
| 33 | $C_{13}H_{11}NO_3$ | 230.1[M+H]$^+$ | 215.0585[M+H-$CH_3$]$^+$ | γ-fagarine or 5-methoxydictamnine |
| | | | 200.0350[M+H-2$CH_3$]$^+$ | fluoroquinoline alkaloids |
| | | | 186.0555[M+H-NH-$CH_3$]$^+$ | |
| | | | 172.0398[M+H-CO-2$CH_3$]$^+$ | |
| | | | 158.0598[M+H-CO-2$CH_3$-$NH_2$]$^+$ | |
| 34* | $C_{15}H_{14}O_5$ | 275.1[M+H]$^+$ | 233.0809[M+H-$CH_3$CO]$^+$ | toddalenone |
| | | | 217.05491[M+H-$CH_3$CO-$CH_4$]$^+$ | |
| | | | 189.0561[M+H-$CH_3$CO-$CH_4$-CO]$^+$ | |
| 35 | $C_{20}H_{18}O_4^+$ | 336.1[M+H]$^+$ | 304.0680[M+H-O$CH_3$-H]$^+$ | berberine |
| | | | 292.0697[M+H-O]$^+$ | |
| | | | 278.0527[M+H-$CH_2$]$^+$ | |
| 36 | $C_{16}H_{18}O_5$ | 291.1[M+H]$^+$ | 219.0660[M+H-CO-$CH_2$-2$CH_3$]+ | aculeatin or toddayanin |
| | | | 205.0501[M+H-CO-2$CH_2$-2$CH_3$]+ | |
| | | | 191.0706[M+H-2CO-$CH_2$-2$CH_3$]+ | |
| 37 | $C_{32}H_{38}O_{10}$ | 583.2[M+H]$^+$ | 566.1922[M+H-OH]$^+$ | toddalin B |
| | | | 291.1233[M+H-$C_{16}H_{18}O_5$]$^+$ | |
| | | | 276.0953[M+H-OH-$C_{16}H_{17}O_5$]$^+$ | |
| | | | 222.0787[M+H-OH-$C_{16}H_{17}O_5$- $C_4H_6$]$^+$ | |
| 38 | $C_{32}H_{40}O_{12}$ | 617.2[M+H]$^+$ | 291.1241[M+H-$H_2O$-$C_{16}H_{20}O_6$]$^+$ | toddalin C |
| | | | 219.0660[M+H-2$CH_3$-$C_{16}H_{20}O_6$-$C_3H_8O$]$^+$ | |
| 39 | $C_{12}H_8O_5$ | 233.3[M+H]$^+$ | 218.0277[M+H-$CH_3$]$^+$ | 5-methoxy-8-hydroxy psoralen |
| | | | 190.0264[M+H-$CH_3$-CO]$^+$ | |
| | | | 162.0324[M+H-$CH_3$-2CO]$^+$ | |
| | | | 147.0068[M+H-$CH_3$-2CO-$CH_3$]$^+$ | |
| 40 | $C_{13}H_{10}O_5$ | 247.1[M+H]$^+$ | 232.0370[M+H-$CH_3$]$^+$ | isopimpinellin |
| | | | 217.0141[M+H-2$CH_3$]$^+$ | |
| | | | 189.0178[M+H-2$CH_3$-CO]$^+$ | |
| | | | 161.0239[M+H-2$CH_3$-2CO]$^+$ | |
| | | | 133.0273[M+H-2$CH_3$-3CO]$^+$ | |
| 41 | $C_{12}H_9NO_2$ | 200.1[M+H]$^+$ | 185.0454[M+H-$CH_3$]$^+$ | dictamnine |
| | | | 129.0568[M+H-$CH_3$-2CO]$^+$ | |
| 42 | $C_{14}H_{13}NO_2$ | 228.1[M+H]$^+$ | 186.0552[M+H-CO-$CH_2$]$^+$ | flindersine |
| | | | 174.0549[M+H-CO-$CH_2$-C]$^+$ | |
| | | | 168.0434[M+H-CO-$CH_2$-$H_2O$]$^+$ | |
| 43 | $C_{15}H_{14}O_4$ | 259.1[M+H]$^+$ | 244.0784[M+H-$CH_3$]$^+$ | 5-methoxyseselin |
| | | | 229.0510[M+H-2$CH_3$]$^+$ | |
| | | | 189.0535[M+H-2CO-$CH_2$]$^+$ | |
| | | | 161.0605[M+H-3CO-$CH_2$]$^+$ | |
| | | | 131.0485[M+H-3CO-$CH_2$-2$CH_3$]$^+$ | |

| Peak | Formula | (+) ESI-MS$^n$ | | Identification |
|---|---|---|---|---|
| | | MS$^n$(Precursor) | Fragment ions | |
| 44 | $C_{32}H_{38}O_{11}$ | 599.2[M+H]$^+$ | 291.1248[M+H-$H_2O$-$C_{16}H_{18}O_5$]$^+$ | toddalin D |
| | | | 219.0654[M+H-2$CH_3$-$C_{16}H_{18}O_5$-$C_3H_8O$]$^+$ | |
| 45* | $C_{16}H_{18}O_5$ | 291.1[M+H]$^+$ | 219.0666[M+H-2CO-$CH_4$]$^+$ | toddanol |
| | | | 205.0527[M+H-2CO-$CH_4$-$CH_2$]$^+$ | |
| | | | 191.0665[M+H-2CO-$CH_4$-2$CH_2$]$^+$ | |
| 46 | $C_{22}H_{30}O_{11}$ | 471.4[M+H]$^+$ | 247.0958[M+H-$CO_2$-$C_6H_{12}O_6$]$^+$ | toddalolactone-3’-*O*-β-d-glucopyranoside |
| | | | 219.0641[M+H-2$CH_3$-$C_9H_{18}O_6$]$^+$ | |
| 47 | $C_{16}H_{20}O_5$ | 293.1[M+H]$^+$ | 221.0720[M+H-$OCH_3$-$CH_2$-CO]$^+$ | peucedanol methyl ether |
| | | | 207.0553[M+H-$OCH_3$-2$CH_2$-CO]$^+$ | |
| 48 | $C_{16}H_{18}O_5$ | 291.1[M+H]$^+$ | 219.0657[M+H-2$CH_3$-$CH_2$-CO]$^+$ | toddanone |
| | | | 205.0521[M+H-2$CH_3$-2$CH_2$-CO]$^+$ | |
| | | | 191.0699[M+H-2$CH_3$-$CH_2$-2CO]$^+$ | |
| 49 | $C_{20}H_{13}NO_5$ | 348.1[M+H]$^+$ | 332.0951[M+H-$CH_4$]$^+$ | oxyavicine |
| | | | 318.0521[M+H-$CH_4$-$CH_2$]$^+$ | |
| | | | 304.0990[M+H-$CH_4$-2$CH_2$]$^+$ | |
| | | | 290.0846[M+H-$CH_4$-2CO]$^+$ | |
| 50 | $C_{23}H_{18}O_7$ | 407.2[M+H]$^+$ | 376.0966[M+H--$OCH_3$]$^+$ | toddacoumaquinone |
| | | | 361.0718[M+H--$OCH_3$-$CH_3$]$^+$ | |
| | | | 333.0774[M+H--$OCH_3$-$CH_3$-CO]$^+$ | |
| | | | 290.0741[M+H--$OCH_3$-$CH_3$-2CO-$CH_3$]$^+$ | |
| 51 | $C_{10}H_8O_4$ | 193.0[M+H]$^+$ | 178.0260[M+H-$CH_3$]$^+$ | scopoletin |
| | | | 150.0309[M+H-$CH_3$-CO]$^+$ | |
| | | | 122.0349[M+H-$CH_3$-2CO]$^+$ | |
| | | | 94.0406[M+H-$CH_3$-3CO]$^+$ | |
| 52 | $C_{21}H_{17}NO_5$ | 364.1[M+H]$^+$ | 349.0960[M+H-$CH_3$]$^+$ | oxynitidine |
| | | | 334.0731[M+H-2$CH_3$]$^+$ | |
| | | | 306.0778[M+H-CO-2$CH_3$]$^+$ | |
| 53 | $C_{21}H_{26}O_5$ | 359.2[M+H]$^+$ | 313.1452[M+H-$CH_3$-$OCH_3$]$^+$ | 8-geranyloxy-5,7-dimethyloxycoumarin |
| | | | 219.0645[M+H-CO--$C_6H_{10}$-2$CH_3$]$^+$ | |
| | | | 233.0476[M+H-CO--$CH_3$-$C_6H_{11}$]$^+$ | |
| | | | 207.0679[M+H-$C_{10}H_{16}O$]$^+$ | |
| 54 | $C_{21}H_{17}NO_5$ | 364.1[M+H]$^+$ | 349.0973[M+H-$CH_3$]$^+$ | oxychelerythrine |
| | | | 334.0734[M+H-2$CH_3$]$^+$ | |
| | | | 306.0783[M+H-CO-2$CH_3$]$^+$ | |
| | | | 291.0549[M+H-CO-3$CH_3$]$^+$ | |
| 55 | $C_{15}H_{15}NO_2$ | 242.2[M+H]$^+$ | 227.0948[M+H-$CH_3$]$^+$ | N-methylflindersine |

| Peak | Formula | (+) ESI-MS$^n$ | | Identification |
|---|---|---|---|---|
| | | MS$^n$(Precursor) | Fragment ions | |
| | | | $226.0876[M+H-CH_4]^+$ | |
| | | | $212.0724[M+H-2CH_3]^+$ | |
| | | | $200.0722[M+H-CH_2-CO]^+$ | |
| 56 | $C_{11}H_{10}O_5$ | $223.1[M+H]^+$ | $208.0368[M+H-CH_3]^+$ | leptodactylone |
| | | | $193.0136[M+H-2CH_3]^+$ | |
| | | | $165.0183[M+H-2CH_3-CO]^+$ | |
| | | | $147.0070[M+H-CH_3-CO_2-OCH_3-CH_3]^+$ | |
| 58 | $C_{16}H_{18}O_5$ | $291.1[M+H]^+$ | $223.0637[M+H-C_5H_8]^+$ | artanin |
| | | | $208.0378[M+H-C_5H_8-CH_3]^+$ | |
| | | | $193.0183[M+H-C_5H_8-2CH_3]^+$ | |
| | | | $178.0263[M+H-C_5H_8-OCH_3-CH_2]^+$ | |
| | | | $165.0184[M+H-C_5H_8-2CH_3-CO]^+$ | |
| 57 | $C_{20}H_{22}O_6$ | $359.2[M+H]^+$ | $293.9495[M+H--3H-CH_4-CO-H_2O]^+$ | matairesinol |
| | | | $223.0619[M+H-C_{12}H_{14}O_4]^+$ | |
| | | | $208.0372[M+H-C_{12}H_{14}O_4-CH_3]^+$ | |
| | | | $137.0589[M+H-C_8H_9O_2\cdot]^+$ | |
| 59 | $C_{16}H_{16}O_4$ | $273.1[M+H]^+$ | $257.0820[M+H-CH_4]^+$ | 6-(3-methyl-1,3-butadienyl)-5,7-dimethoxycoumarin |
| | | | $243.0665[M+H-2CH_3]^+$ | |
| | | | $217.0601[M+H-C_2H_2]^+$ | |
| | | | $161.0594[M+H-2CH_3-2CO-C_2H_2]^+$ | |
| 60 | $C_{15}H_{14}O_4$ | $259.1[M+H]^+$ | $244.0728[M+H-CH_3]^+$ | 6-methoxyseselin |
| | | | $215.0365[M+H-CH_4-CO]^+$ | |
| | | | $217.0502[M+H-CO-CH_2]^+$ | |
| | | | $229.0496[M+H-2CH_3]^+$ | |
| | | | $189.0540[M+H-2CO-CH_2]^+$ | |
| | | | $161.0597[M+H-3CO-CH_2]^+$ | |
| 61 | $C_{17}H_{16}O_5$ | $301.1[M+H]^+$ | $233.0455[M+H-C_5H_8]^+$ | phellopterin |
| | | | $230.0214[M+H-CH_3-2CO]^+$ | |
| | | | $218.0218[M+H-C_5H_8-CH_3]^+$ | |
| | | | $217.0140[M+H-CH_3-C_5H_9]^+$ | |
| | | | $190.0259[M+H-CH_3-CO-C_5H_8]^+$ | |
| | | | $162.0310[M+H-CH_3-2CO-C_5H_9]^+$ | |
| 62 | $C_{16}H_{18}O_4$ | $275.1[M+H]^+$ | $219.0661[M+H-C_4H_8]^+$ | coumurrayin |
| | | | $204.0456[M+H-C_4H_9-CH_3]^+$ | |
| | | | $176.0514[M+H-C_4H_9-CH_3-CO]^+$ | |
| | | | $131.0489[M+H--C_4H_9-CH_4-CO-CO_2]^+$ | |
| 63* | $C_{16}H_{16}O_4$ | $273.1[M+H]^+$ | $243.0658[M+H-2CH_3]^+$ | *cis*-dehydrocoumurrayin |
| | | | $219.0669[M+H-2CH_3-C_2H_2+2H]^+$ | |

| Peak | Formula | (+) ESI-$MS^n$ | | Identification |
|---|---|---|---|---|
| | | $MS^n$(Precursor) | Fragment ions | |
| | | | 189.0550[$M+H-C_4H_6-CH_2-CH_3$]$^+$ | |
| | | | 161.0594[$M+H-C_4H_6-CH_2-CH_3-CO$]$^+$ | |
| 64* | $C_{20}H_{16}NO_4^+$ | 334.1[M+H]$^+$ | 319.0861[$M+H-CH_3$]$^+$ | *O*-demethylnitidine |
| | | | 304.0611[$M+H-2CH_3$]$^+$ | |
| | | | 291.0910[$M+H-CH_3-CO$]$^+$ | |
| | | | 276.0668[$M+H-2CH_3-CO$]$^+$ | |
| 65 | $C_{16}H_{18}O_4$ | 275.1[M+H]$^+$ | 245.0726[$M+H-2CH_3$]$^+$ | 8-(3,3-dimethylallyl)-6,7-dimethoxycoumarin |
| | | | 163.0647[$M+H-4CH_3-2CO-C_2H_2$]$^+$ | |
| | | | 221.0707[$M+H-2CO$]$^+$ | |
| 66 | $C_{14}H_{12}O_4$ | 245.1[M+H]$^+$ | 229.0878[$M+H-CH_4$]$^+$ | norbraylin |
| | | | 201.0564[$M+H-CH_4-CO$]$^+$ | |
| | | | 215.0712[$M+H-2CH_3$]$^+$ | |
| | | | 187.0394[$M+H-2CH_3-CO$]$^+$ | |
| | | | 159.0426[$M+H-2CH_3-2CO$]$^+$ | |
| 67 | $C_{13}H_{11}NO_4$ | 246.1[M+H]$^+$ | 188.0432[$M+H-NH-O-2CH_2$]$^+$ | haplopine |
| | | | 132.0520[$M+H-NH-O-2CH_2-CO$]$^+$ | |
| 68* | $C_{16}H_{18}O_4$ | 275.1[M+H]$^+$ | 260.0951[$M+H-CH_3$]$^+$ | toddaculin |
| | | | 245.0805[$M+H-OCH_3$]$^+$ | |
| | | | 221.0707[$M+H-C_4H_6$]$^+$ | |
| | | | 205.0495[$M+H-C_4H_6-CH_3$]$^+$ | |
| | | | 161.0591[$M+H-C_4H_6-CH_3-HCOO-$]$^+$ | |
| 69 | $C_{21}H_{30}O_4$ | 347.2[M+H]$^+$ | 329.2138[$M+H-H_2O$]$^+$ | nelumol A |
| | | | 193.0868[$M+H-C_{10}H_{17}OH$]$^+$ | |
| | | | 161.0604[$M+H-H_2O-C_{10}H_{18}-2CH_3$]$^+$ | |
| 70 | $C_{20}H_{15}NO_4$ | 334.1[M+H]$^+$ | 318.0776[$M+H-CH_4$]$^+$ | dihydroavicine |
| | | | 290.0825[$M+H-CO-CH_4$]$^+$ | |
| | | | 276.0668[$M+H-CO-CH_4$]$^+$ | |
| 71 | $C_{21}H_{19}NO_4$ | 350.1[M+H]$^+$ | 335.1176[$M+H-CH_3$]$^+$ | dihydronitidine |
| | | | 319.0866[$M+H-CO-CH_3-CH_4$]$^+$ | |
| | | | 305.074[$M+H-CO-CH_3-CH_4-CH_2$]$^+$ | |
| | | | 304.0988[$M+H-CO-CH_3-CH_4-CH_2-H$]$^+$ | |
| | | | 290.0824[$M+H-CO-CH_3-CH_4-2CH_2-H$]$^+$ | |
| 72 | $C_{32}H_{34}O_9$ | 563.3[M+H]$^+$ | 273.1146[$M+H-C_{11}H_8O_4-CO-2CH_2-2CH_3$]$^+$ | toddalosin |
| | | | 219.0662[$M+H-C_{11}H_8O_4-CO-2CH_2-2CH_3-C_4H_6$]$^+$ | |
| 73-76, 78 | $C_{32}H_{32}O_8$ | 545.2[M+H]$^+$ | 219.0654[$M+H-C_{20}H_{22}O_4$]$^+$ | mexolide |

| Peak | Formula | (+) ESI-$MS^n$ | | Identification |
|---|---|---|---|---|
| | | $MS^n$(Precursor) | Fragment ions | |
| 77 | $C_{30}H_{26}O_8$ | 515.3$[M+H]^+$ | $[M+H]^+$ at m/z 515 indicated a dimeric coumarin with a dimethyl pyran and one methoxyl group both on ring A | toddasiatin |

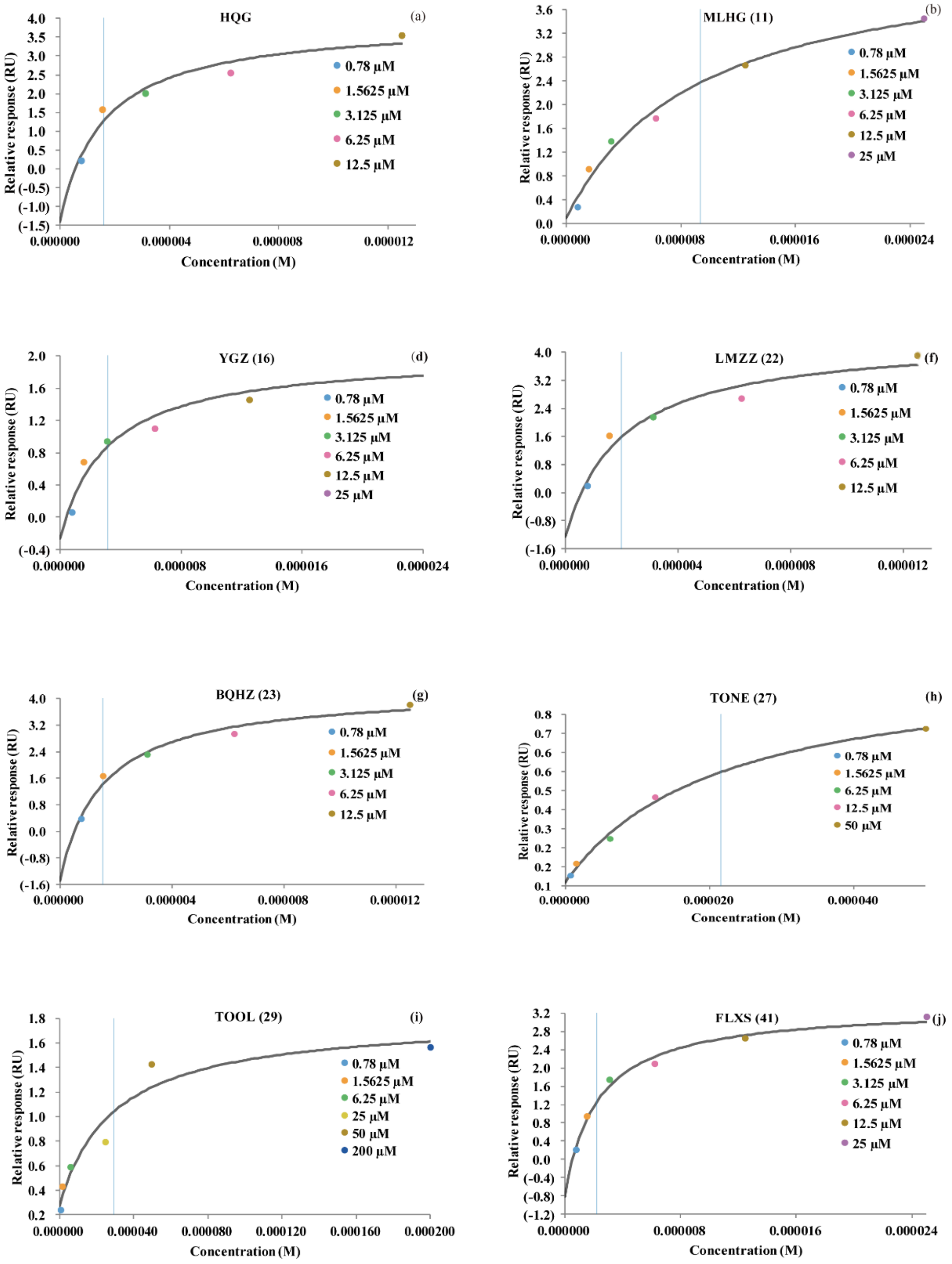


**Figure S2.** PD-L1 binding activity validation results in affinity models of the potential compounds from TA by SPR. (a) Affinity of HQG and PD-L1; (b) affinity of MLHG (**11**) and PD-L1; (d) affinity of YGZ (**15**) and PD-L1; (f) affinity of LMZZ (**22**) and PD-L1; (g) affinity of BQHZ (**23**) and PD-L1; (h) affinity of TONE (**27**) and PD-L1; (i) affinity of TOOL (**29**) and PD-L1; (j) affinity of FLXS (**41**) and PD-L1.

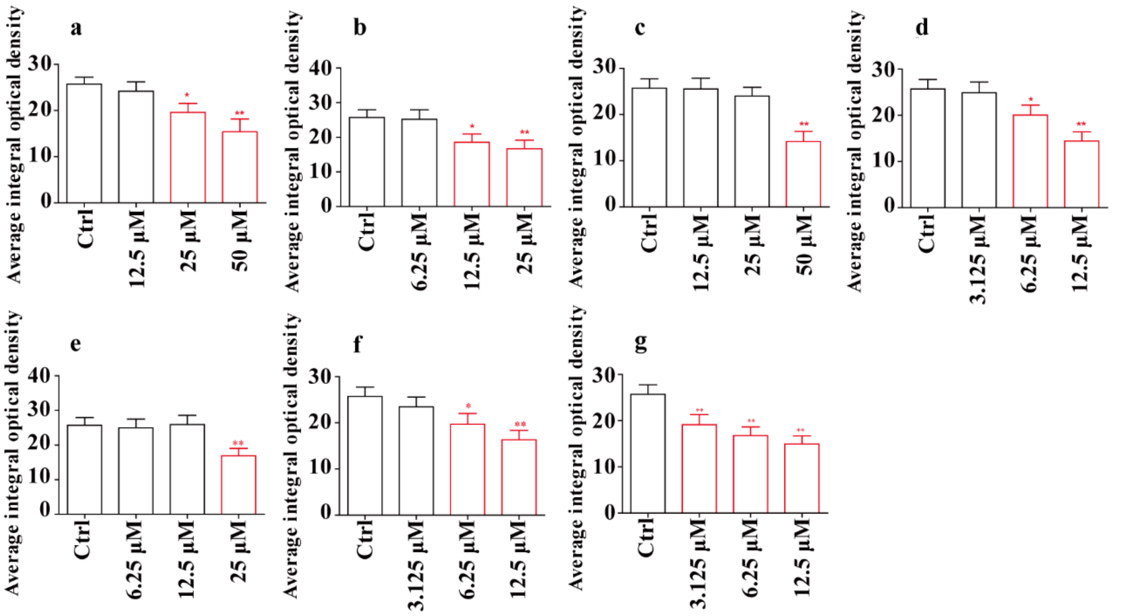


**Figure S3.** Effects of MLHG (a), LMZZ (b), BQHZ (c), FLXS (d), YGZ (e), TOOL (f), and HQG (g) on the interaction of PD-1 Fc and in PD-L1 A549 cells. After A549 cells incubated in the presence of a (MLHG, 12.5, 25, 50 µM), b (LMZZ, 6.25, 12.5, 25 µM), c (BQHZ, 12.5, 25, 50 µM), d (FLXS, 3.125, 6.25, 12.5 µM), e (YGZ, 6.25, 12.5, 25 µM), f (TOOL, 3.125, 6.25, 12.5 µM), and g (HQG, 3.125, 6.25, 12.5 µM) for 24 h, microscopy images show the association of PD-L1 on A549 cell membranes with PD-1/Fc protein. Green fluorescence (Alexa Fluor® 488-labeled PD-1/Fc protein) indicates the conjugated PD-1 Fc nucleus was stained by DAPI (blue fluorescence). Results were mean ± SD for three individual experiments, which, for each condition, were performed in triplicate. The statistical histogram of integral optical density of several experimental and control groups was presented. Results were mean ± SD for three individual experiments, which, for each condition, were performed in triplicate. * $P < 0.05$, ** $P < 0.01$.

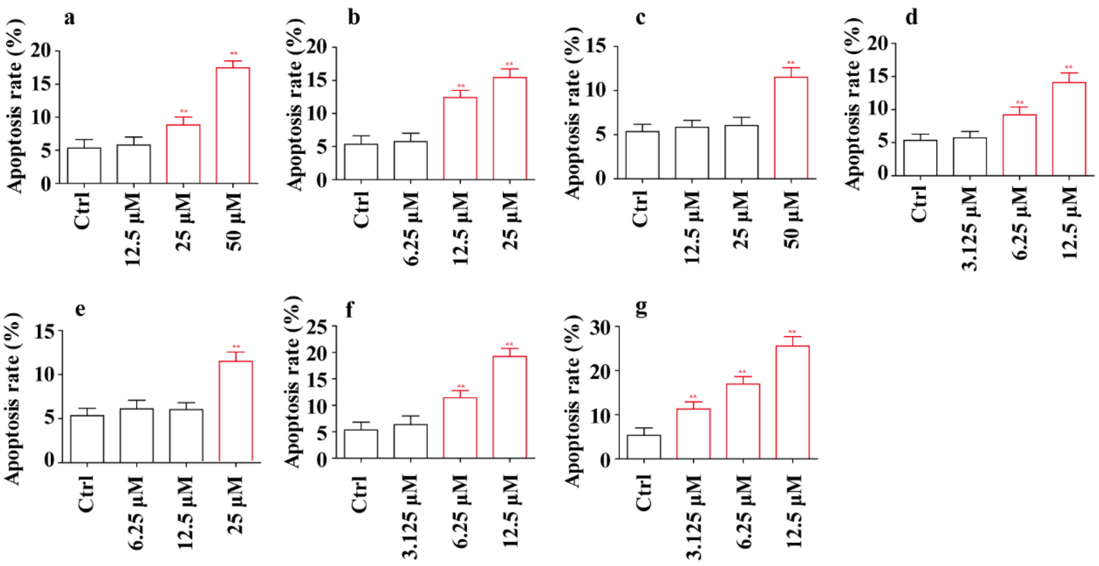


**Figure S4.** The statistical histogram of apoptosis rate of MLHG (a), LMZZ (b), BQHZ (c), FLXS (d), YGZ (e), TOOL (f), and HQG (g). Results are means ± SD for three individual experiments which, for each condition, were performed in triplicate. * $P < 0.05$, ** $P < 0.01$.

**References**

**1.** Cao, Y.; Fang, J.; Shi, Y.; Wang, H.; Chen, X.; Liu, Y.; Zhu, Z.; Cao, Y.; Hong, Z.; Chai, Y., Screening potential P-glycoprotein inhibitors by combination of a detergent-free membrane protein extraction with surface plasmon resonance biosensor. Acta Pharm Sin B. **2022** Jul; *12*(7): 3113-3123.
